# Effect of Universal Adhesives on Resin Cement–Fiber Post–Core Materials

**DOI:** 10.3390/polym18070810

**Published:** 2026-03-26

**Authors:** Masao Irie, Masahiro Okada, Yukinori Maruo, Kenraro Akiyama, Kumiko Yoshihara, Akimasa Tsujimoto, Takuya Matsumoto

**Affiliations:** 1 Department of Biomaterials, Graduate School of Medicine, Dentistry and Pharmaceutical Sciences, Okayama University, 2-5-1, Shikata-cho, Kita-ku, Okayama 700-8525, Japan; tmatsu@md.okayama-u.ac.jp; 2 Department of Dental Biomaterials, Graduate School of Dentistry, Tohoku University, 4-1 Seiryo-machi, Aoba-ku, Sendai 980-8575, Japan; masahiro.okada.c2@tohoku.ac.jp; 3 Department of Prosthodontics, Okayama University, 2-5-1, Shikata-cho, Kita-ku, Okayama 700-8558, Japan; ykmar@md.okayama-u.ac.jp; 4 Department of Occlusal and Oral Functional Rehabilitation, Graduate School of Medicine, Dentistry and Pharmaceutical Sciences, Okayama University, 2-5-1 Shikata-cho, Kita-ku, Okayama 700-8525, Japan; akentaro@md.okayama-u.ac.jp; 5 Health Research Institute, National Institute of Advanced Industrial Science and Technology, 2217-14 Hayashi-cho, Takamatsu 761-0395, Japan; kumiko.yoshihara@aist.go.jp; 6 Department of Operative Dentistry, School of Dentistry, Aichi Gakuin University, Nagoya 700-8558, Japan; aki-tj@dpc.agu.ac.jp

**Keywords:** bonding performance, universal adhesive, fiber post, luting materials, root dentin

## Abstract

This study evaluated eleven resin cements used as core build-up materials by examining the following properties: (a) push-out force between root dentin and the fiber post; (b) pull-out force between the fiber post and the core build-up material; (c) shear bond strength of the resin cement to root dentin; (d) flexural strength of the resin cement; and (e) flexural modulus of elasticity of the resin cement. The purpose of this investigation was to clarify the relationships between recently available universal adhesives, core build-up materials, resin cements, and fiber posts. All experiments were performed at two evaluation periods: after 1 day of water storage (Base) and after 20,000 thermocycles (TC 20k). For the push-out test, simulated post spaces were prepared in single-rooted human premolars. The specimens were sectioned perpendicular to the long axis into 2 mm-thick slices and then subjected to push-out testing to assess the bond strength of the dentin–resin cement–fiber post complex. No significant differences in bonding performance were found between Base and TC 20k. These findings suggest that universal adhesives used for pretreatment of multiple substrates in fiber post cementation can provide not only strong but also durable adhesion over time.

## 1. Introduction

In contemporary restorative dentistry, achieving a natural appearance and a confident smile are often regarded as essential treatment goals. For this reason, fiber posts combined with resin core build-ups are widely used in esthetically demanding restorations. Unlike conventional cast metal posts, fiber posts exhibit an elastic modulus closer to that of dentin, which helps distribute stress more favorably and lowers the risk of root fracture after endodontic treatment [1,2,3,4,5,6]. When endodontically treated teeth lack a sufficient ferrule, the missing coronal structure can be reconstructed with a resin core. Nevertheless, this procedure may become technique-sensitive, because adhesion must be established to dissimilar substrates. In such situations, the cured composite resin used to replace the ferrule generally requires silane treatment, whereas the remaining root dentin is typically conditioned with an acid monomer-containing agent [7,8,9,10].

Recently developed resin cements have overcome the major hurdle of requiring different surface treatment agents for different substrates when used as a core build-up material. These updated resin cements can be applied to all types of substrates and are shown to exhibit strong adhesion to root canal dentin. In addition, they no longer require one surface treatment agent for root canal dentin and a different one for the cured composite resin (restored portion of the ferrule). Other improvements which favor their pragmatic use as a core build-up material include possessing excellent mechanical strength to withstand occlusal bite forces during mastication [11,12,13,14].

Fiber posts are typically reinforced with E-glass or S-glass fibers. Known for their high tensile strength, excellent flexibility and biocompatibility, these fibers are embedded within a polymeric resin matrix, typically epoxy or methacrylate polymer, to create a composite material with an elastic modulus ranging from 16 to 40 GPa [4]. In addition to their esthetic appeal, fiber posts offer several advantages such as high impact resistance, high shock absorption capacity, increased fatigue resistance, and reduced root fracture risk [15]. In endodontically treated teeth, fiber posts are traditionally bonded to the root canal surface using resin cement and a silane primer, thereby forming an integrated dentin–post adhesive system.

A newer generation of adhesive materials, referred to as universal adhesives, has recently been introduced into clinical practice. These systems are designed to simplify bonding procedures by allowing a one-step application to tooth substrates. In addition to their use on dental tissues, universal adhesives can also promote bonding to a range of restorative materials, including zirconia, metals, and several ceramic substrates, often without the need for a separate priming step [16,17,18,19].

Our previous studies examined how the flexural characteristics of the resin cements used for core build-up influence bonding behavior. Specifically, we assessed whether the flexural strength and flexural modulus of these materials were associated with their shear bond strength to root dentin and with the retention of indirect restorative components, such as dental posts and lithium disilicate ceramic restorations [18,19]. More recently, our attention shifted toward the role of universal adhesives in the long-term stability of bonded interfaces. Under conditions without hydrofluoric acid (HF) pretreatment, Tokuyama Universal Bond II demonstrated significantly greater shear bond strength to lithium disilicate (LDS) ceramic than other bonding agents after 20,000 thermocycles [19]. These findings suggested that, for LDS, durable long-term bonding may be achieved with Tokuyama Universal Bond II even without the use of hazardous HF.

With respect to post retention, the relationship between pull-out force and push-out force has not yet been sufficiently clarified in the literature. This lack of direct evidence led us to undertake the present study using currently available resin cements.

The present study evaluated a simplified cementation approach in which universal adhesives were used with fiber posts, root dentin, and recently introduced core build-up materials. Measurements were obtained at two time points: after 1 day of storage (Base) and after 20,000 thermocycles (TC 20k). The assessed outcomes related to fiber post cementation were as follows: (a) push-out force at the interface between root dentin and the fiber post; (b) pull-out force between the fiber post and the core build-up material; (c) shear bond strength of the resin cement to root dentin; (d) flexural strength of the resin cement; and (e) flexural modulus of the resin cement.

This study was designed to test three hypotheses. First, value (a) would show correlations with one or more of the following parameters: (b), (c), (d), and (e). Second, universal adhesives would produce durable bonding between root dentin, fiber posts, and core build-up materials, with no significant influence of the evaluation period (Base vs. TC 20k). Third, Tokuyama Universal Bond II would demonstrate effectiveness as a universal adhesive across different core build-up materials, luting cements, and fiber posts.

## 2. Materials and Methods

The materials used in this study, together with their manufacturers and compositions, are summarized in Table 1, Table 2 and Table 3.

Ten specimens were prepared for each resin cement in each test condition and at each evaluation period. All experimental procedures were carried out by a single operator in accordance with the manufacturers’ instructions. A light-curing unit (G-Light Prima II; GC, Tokyo, Japan) was used for photoactivation, and its irradiance was verified with a radiometer (Demetron; Kerr, Danbury, CT, USA) before each application. Throughout the experiments, the output was maintained at 450 mW/cm^2^. Human premolars and molars extracted for orthodontic purposes served as the tooth specimens. After extraction, each tooth was immediately placed in cold distilled water and stored at approximately 4 °C for 1 to 2 months until use. The study protocol was approved by the Ethics Committee of Okayama University Graduate School of Medicine, Dentistry and Pharmaceutical Sciences and Okayama University Hospital (No. 1901-036; Elucidation of Caries Pathology Using Extracted Teeth and Its Application to Treatment Methods; 2 June 2019).

### 2.1. Push-Out Test

Simulated cavities were prepared using extracted single-rooted human premolars with K reamers (new Endo K Reamers, Shofu, Kyoto, Japan; 25 mm length, Sizes 10 and 15), K-files (new Endo K-Files, Shofu, Kyoto, Japan; 25 mm length, Sizes 25 and 30) and a Peeso reamer (new Endo Peeso Reamer, Shofu, Kyoto, Japan; 16 mm length, Size 6) according to conventional methods. Root canals of the simulated cavities were cleaned with a 3–6% sodium hypochlorite solution (Antiformin, Nippon Shika Yakuhin, Shimonoseki, Japan) for 1 min, followed by a 3% EDTA solution (SMEARCLEAN, Nippon Shika Yakuhin, Shimonoseki, Japan) for 1 min.

Root dentin surfaces were pretreated with universal adhesives according to each manufacturer’s instructions (Table 2).

Each fiber post surface (Table 3) was conditioned with the universal adhesive assigned to the corresponding system, following the manufacturers’ instructions provided in Table 2. For Monobond Plus (Ivoclar Vivadent) and OptiBond eXTRa Universal (Kerr Dental), no specific post system was recommended by the manufacturers; therefore, BeautiCore FiberPost (diameter, 1.6 mm; Shofu, Kyoto, Japan; Table 3) was selected for use in these groups.

**Figure 1:**
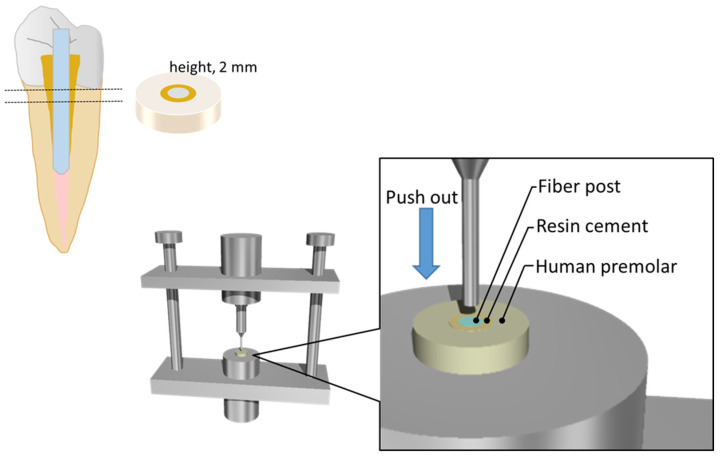
Schematic illustration of push-out test.

For each system listed in Table 1, the corresponding resin cement paste was introduced into the prepared post space, after which the pretreated fiber post was inserted into the root canal. Polymerization was achieved by two 20 s light irradiations delivered from the occlusal aspect of the post with a light-curing unit (G-Light Prima II; GC, Tokyo, Japan). The resulting specimens were then stored in water at 37 °C for 24 h. After storage, the coronal portion of each specimen was removed with a low-speed precision cutter (IsoMet; Buehler, Lake Bluff, IL, USA). Each root specimen was subsequently sectioned perpendicular to its long axis into 2 mm-thick slices (Figure 1). These specimens were used to evaluate the bond strength within the integrated dentin–post adhesive complex, consisting of human dentin, the intervening resin cement layer, and the fiber post.

All failed specimens were checked with a light microscope (SMZ-10, Nikon, Tokyo, Japan) to determine the nature of their fractures [18,19].

### 2.2. Pull-Out Test

Each post was pretreated with a universal adhesive according to the manufacturers’ instructions in Table 2. For Ivoclar Vivadent’s Monobond Plus and Kerr’s OptiBond eXTRa Universal adhesives, which had no instructed post procedure system, BeautiCore FiberPost (Diameter: 1.6 mm; Shofu, Kyoto, Japan; Table 3) was utilized as the post procedure system.

Each resin core build-up material was filled in a Teflon mold (upper diameter: 8 mm, bottom diameter: 3.6 mm, and height: 5 mm) set on a glass plate pre-coated with Vaseline. Then, each post was inserted at the center of the Teflon mold using a retainer and cured in four overlapping sections, with each section cured for 20 × 4 s, as shown in Figure 1

The specimens thus obtained were mounted on a universal testing machine (5565, Instron, Canton, MA, USA), and pull-out force was applied at a crosshead speed of 0.5 mm/min (*n* = 10/group), as shown in Figure 1. As each post differed in its external form, the maximum failure load was expressed in Newton (N). After the pull-out force tests, all failed specimens were checked with a light microscope (50×; Measurescope MM-II, Nikon, Tokyo, Japan) to determine the status of their fracture modes. Three categories of fractured mode were evaluated: (1) adhesive failure at the interface between post and resin core material; (2) cohesive failure within the resin core material; and (3) combination of adhesive and cohesive failures on the same surface or a mixed failure [19].

### 2.3. Shear Bond Strength of Resin Cement to Root Dentin

Human premolars and molars were used to prepare the specimens. Each tooth was embedded in a slow-polymerizing epoxy resin (EpoFix Resin; Struers, Copenhagen, Denmark) with the buccal surface oriented upward. The exposed surface was then wet ground with silicon carbide abrasive papers up to 320 grit until a superficial dentin area of at least 4 mm in diameter was obtained. A split Teflon mold containing a cylindrical cavity (diameter, 3.6 mm; height, 2 mm) was secured to the prepared dentin surface using a mounting apparatus. After the corresponding dentin treatment agent had been applied according to the manufacturers’ instructions shown in Table 2, the mold cavity was filled with the resin cement paste. The material was then light-cured for 20 s from each of the two directions. Following storage in distilled water at 37 °C for 24 h, the specimens were subjected to shear loading with a universal testing machine (Autograph DCS-2000; Shimadzu, Kyoto, Japan) at a crosshead speed of 0.5 mm/min. Load was applied perpendicular to the bonded interface through a flat, blunt shearing blade 1 mm in thickness. The stress at failure was calculated and recorded as the shear bond strength. Fractured specimens were subsequently examined under a light microscope (SMZ-10; Nikon, Tokyo, Japan) to determine the mode of failure [18,19,20].

### 2.4. Flexural Strength and Flexural Modulus of Elasticity of Resin Cement

Specimens for flexural testing were prepared from each resin cement (*n* = 10 per group) using a Teflon mold measuring 25 mm × 2 mm × 2 mm. Each paste was light-cured in three overlapping segments for 20 s per segment, and the opposite side of the specimen was irradiated in the same manner. After storage in distilled water at 37 °C for 24 h, the specimens were subjected to a three-point bending test with a span length of 20 mm at a crosshead speed of 0.5 mm/min using a universal testing machine (5565; Instron, Canton, MA, USA), in accordance with ISO 9917-2:1998 [21]. Flexural strength and flexural modulus of elasticity were then calculated with the Series IX software v8.27 (Instron, Canton, MA, USA).

### 2.5. Scanning Electron Microscope (SEM) Observations

After the pull-out test, some fractured specimens were randomly chosen for surface observation by scanning electron microscopy (SEM; JSM-IT800 SHL, Jeol, Tokyo, Japan). The microscope was operated at an accelerating voltage of 5 kV. Before SEM examination, the specimen surfaces were coated with a thin layer of osmium using an osmium coater (Neoc-STB, Meiwafosis, Tokyo, Japan) to minimize surface charging.

### 2.6. Statistical Analysis

Statistical analyses were performed using Statistica 9.1 (StatSoft, OK, USA) and SPSS version 19 (Chicago, IL, USA). Data for push-out force, pull-out force, shear bond strength to root dentin, flexural strength, and flexural modulus of elasticity were analyzed by two-way analysis of variance (ANOVA), followed by Tukey’s honestly significant difference (HSD) test for post hoc multiple comparisons. Correlations of push-out force with pull-out force, shear bond strength to root dentin, flexural strength, and flexural modulus of elasticity were assessed using Spearman’s correlation test. In addition, multiple linear regression analysis was performed on the basis of the correlation results to further examine the relationships among push-out force, pull-out force, and flexural modulus of elasticity. The significance level was set at *p* < 0.05.

## 3. Results

### 3.1. Push-Out Test

The push-out force values and the results of the corresponding statistical analyses are presented in Table 4.

For most post–core systems, no significant change in push-out force was detected over time (*p* > 0.05), with the exception of the MultiCore Flow and NX3 systems. In general, higher mean values were recorded after 1 day of storage than after TC 20k. Among the tested systems, the SI-300381 system showed the highest push-out force at both evaluation periods, whereas the MultiCore Flow system consistently produced lower values.

Regarding failure patterns, no purely adhesive failure was identified. Overall, the distribution of failure modes was comparable between the two time periods across all systems.

### 3.2. Pull-Out Test for Pretreatment with Manufacturers’ Recommended Agents

The pull-out force values and the results of the statistical analyses are presented in Table 5.

For most post–core systems, pull-out force did not change significantly over time (*p* > 0.05), except for the MultiCore Flow and NX3 systems. In general, higher mean values were observed after 1 day of storage than after TC 20k, with the exception of UniFil Core EM. BeautiLink SA Automix exhibited the highest pull-out force at both evaluation periods. On failure mode, no adhesive fractures were observed. Overall, the proportion of adhesive failures was the same at both time periods. With regard to failure mode, no purely adhesive failure was detected. Overall, the distribution of failure modes was similar between the two time periods.

### 3.3. Pull-Out Test for Pretreatment with Tokuyama Universal Bond II

The pull-out force values and the corresponding statistical results for when Tokuyama Universal Bond II was used as the only pretreatment agent are presented in Table 6.

For most post–core systems, pull-out force did not change significantly over time (*p* > 0.05), although significant differences were found for the UniFil Core EM, MultiCore Flow, and NX3 systems. Notably, all systems maintained values above 30 MPa even after TC 20k.

No purely adhesive failure was observed in any group. In addition, the distribution of failure modes was similar between the two evaluation periods. As shown in Table 6, pretreatment with Tokuyama Universal Bond II resulted in higher pull-out force values across all post–core systems at both time points. These findings indicate that the bonding performance of Tokuyama Universal Bond II was effective not only within its own system but also across post–core systems from other manufacturers.

### 3.4. Statistical Comparisons Between Two Pretreatment Agents

After pretreatment with Tokuyama Universal Bond II, all post–core systems evaluated in this study maintained values above 30 MPa even after TC 20k. A significant difference between the two pretreatment protocols was detected only in cases where the manufacturer-recommended agent produced values below 30 MPa (Table 7).

### 3.5. Shear Bond Strength to Root Dentin

The shear bond strength values and the corresponding statistical results are presented in Table 8.

For most core build-up materials, no significant change in shear bond strength was observed over time (*p* > 0.05), except for NX3 and BeautiLink SA Automix. In general, higher mean values were recorded after 1 day of storage than after TC 20k, with the exception of MultiCore Flow. Among the materials tested, Clearfil DC Core Automix ONE showed the highest values at both evaluation periods.

No purely adhesive failure was observed in any group. Overall, the distribution of failure modes was similar between the two time periods.

### 3.6. Flexural Strength

The flexural strength values of the core build-up materials and the corresponding statistical results are presented in Table 9.

In general, higher mean flexural strength values were obtained after 1 day of storage than after TC 20k. Approximately half of the materials showed a statistically significant reduction after 20,000 thermocycles (*p* < 0.05). Among the core build-up materials tested, ESTECORE Hand Type exhibited the highest flexural strength at both evaluation periods.

### 3.7. Flexural Modulus of Elasticity

The flexural modulus of elasticity values of the core build-up materials and the corresponding statistical results are presented in Table 10.

Nearly all core materials showed no statistically significant decrease after TC 20k (*p* > 0.05). As observed for flexural strength, ESTECORE Hand Type exhibited the highest values at both evaluation periods.

### 3.8. SEM Observations

**Figure 2:**
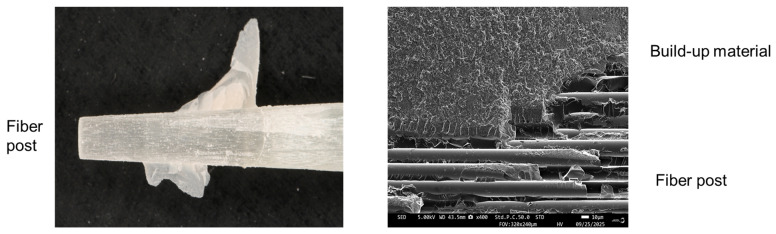
Left: Close-up image of core build-up material adhering to the fiber post. Right: SEM image of core build-up material adhering firmly to the fiber post and penetrating the fibers.

Representative SEM images of fractured surfaces after pull-out testing are shown in Figure 2.

The lower-magnification SEM images showed a cohesive failure pattern at the interface between the post (Tokuyama FR Post) and core material (ESTECEM II Plus). In other words, the post surface was chemically bonded to the core build-up material through universal adhesive pretreatment.

### 3.9. Correlations

**Figure 3:**
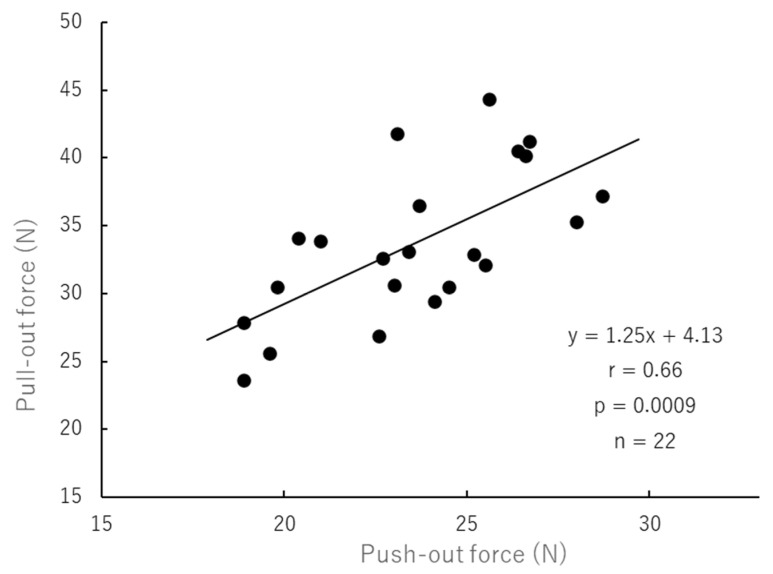
Relationship between push-out force and pull-out force.

The relationships among the tested variables for all core build-up materials at both evaluation periods (*n* = 22) are summarized in Table 11. Push-out force was significantly correlated with pull-out force (r = 0.656, *p* = 0.0009; Figure 3) and with flexural modulus of elasticity (r = 0.475, *p* = 0.025; Table 11).

**Figure 4:**
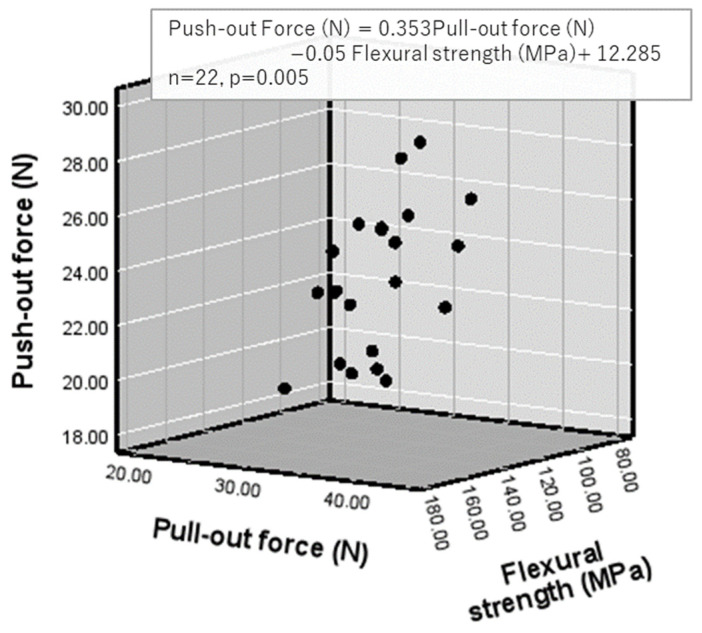
Relationships among push-out force, pull-out force and flexural strength.

Multiple linear regression analysis further yielded the following equations:

Push-out force = 0.353 × pull-out force − 0.05 × flexural strength + 12.285 (*p* = 0.005; Figure 4).

**Figure 5:**
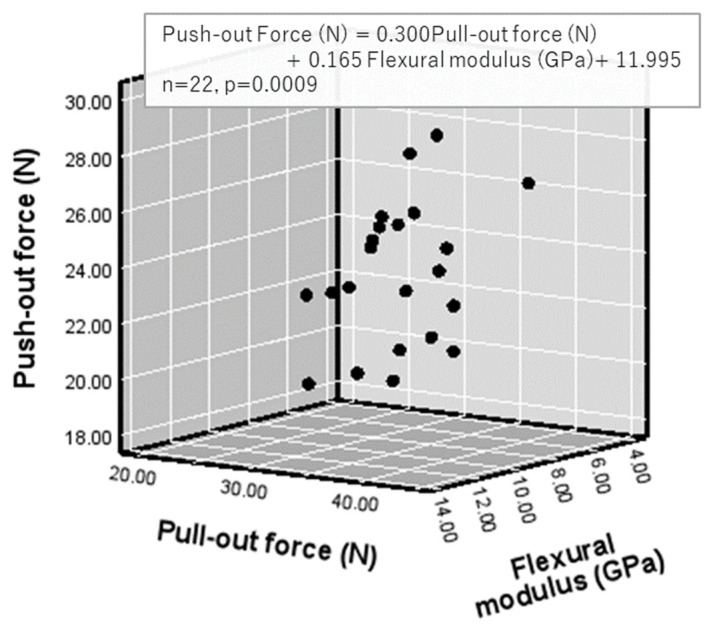
Relationships among push-out force, pull-out force and flexural modulus.

Push-out force = 0.300 × pull-out force + 0.165 × flexural modulus + 11.995 (*p* = 0.0009; Figure 5).

## 4. Discussion

The post and core system comprises the root canal dentin surface, core build-up material and the post surface, thereby producing two adhesive interfaces. Given the strong trend towards the use of fiber posts and resin cores for tooth restorations, a diverse range of products from a myriad of manufacturers have emerged to cater to this unabating demand. Therefore, the long-term durability of fiber post cementation should not just focus on the versatility and ease of use of universal adhesives for multiple substrates within the two adhesive interfaces, it should further explore the possibility of a singular, general-purpose universal adhesive that could be applied on other manufacturers’ post and core systems to yield the same effective bonding.

In this study, the influence of universal adhesives on fiber post cementation with resin core materials was evaluated through measurements of push-out force, pull-out force, shear bond strength, flexural strength, and flexural modulus of elasticity. In a separate analysis, Tokuyama Universal Bond II was also applied to post–core systems from other manufacturers in order to assess its bonding effectiveness across different materials.

### 4.1. Correlation Between Push-Out Force and Pull-Out Force (Table 11 and Figure 3)

In the present study, push-out force showed a strong positive correlation with pull-out force (r = 0.656, *p* = 0.0009, y = 1.25x + 4.13; *n* = 22). This finding is reasonable because both tests were performed using the same combinations of pretreatment agents, fiber posts, and core build-up materials. Although push-out and pull-out loading represent different testing configurations, both reflect the integrity of bonding within the post–core complex. The observed association therefore suggests that factors contributing to improved interfacial retention in one test condition may also enhance resistance under the other.

### 4.2. Correlation Between Push-Out Force and Flexural Modulus of Elasticity or Flexural Strength (Table 11 and Figure 4)

In the present study, flexural strength of the core build-up materials was not significantly correlated with push-out force, although a tendency toward correlation was observed (r = 0.409, *p* = 0.058). By contrast, flexural modulus of elasticity showed a significant correlation with push-out force (r = 0.475, *p* = 0.025). These findings suggest that resistance to post displacement during push-out testing is influenced more by material stiffness than by ultimate flexural strength. In other words, the ability of the bonded assembly to resist deformation appears to be more relevant to push-out behavior than the maximum strength of the core material itself.

Elastic modulus represents the stiffness of a material within its elastic deformation range. Because natural tooth tissues also possess characteristic modulus values, combining them with restorative materials of dissimilar stiffness may alter the mechanical behavior of the restored tooth and generate interfacial stress. For this reason, compatibility of elastic modulus between dentin and core build-up materials is considered important for reducing stress concentration produced by thermal changes, occlusal loading, or polymerization shrinkage. From this perspective, core build-up materials with a relatively high elastic modulus closer to that of dentin may contribute to more favorable biomechanical performance in post–core restorations [3].

### 4.3. Correlation Between Push-Out Force and Shear Bond Strength (Table 11)

Push-out force did not correlate with shear bond strength (*p* > 0.10). After push-out testing, only a small amount of core material adhered to the post. Since the majority of the root canal wall–core build-up material interface showed clear delamination failure, the effect of tooth structure adhesion appeared minimal, suggesting no correlation. This result could be due to pretreatment by universal adhesives. It was possible that the adhesive systems marketed by various manufacturers as “universal adhesives” failed to achieve the strong bond required for root canal dentin, such as that needed for bonding to core build-up materials or fiber posts.

### 4.4. Correlations Among Push-Out Force, Pull-Out Force, and Flexural Strength or Flexural Modulus of Elasticity (Figure 4 and Figure 5)

Both push-out force and pull-out force were associated with the flexural properties of the core build-up materials. Multiple regression analysis showed significant relationships for models incorporating flexural strength (Push-out Force (N) = 0.353 × Pull-out Force (N) − 0.05 × Flexural Strength (MPa) + 12.285, *n* = 22, *p* = 0.005) and flexural modulus of elasticity (Push-out Force (N) = 0.300 × Pull-out Force (N) + 0.165 × Flexural Modulus (GPa) + 11.995, *n* = 22, *p* = 0.0009). These findings indicate that the mechanical properties of the core build-up materials, particularly their stiffness, contribute to the retentive behavior between root canal dentin and fiber posts (Figure 2). They also support the first hypothesis, although no correlation was found between push-out force and shear bond strength to root dentin.

The present results further suggest that push-out performance may be estimated, at least in part, from the flexural properties of the core build-up material, especially the flexural modulus of elasticity. From a practical standpoint, this raises the possibility of screening material performance without relying exclusively on experiments using human premolars. In this sense, the findings provide partial support for the second hypothesis. When core build-up materials with favorable stiffness characteristics are combined with universal adhesives, durable bonding may be achieved throughout the post–core system.

However, retention is not determined solely by the intrinsic properties of the core material. The surrounding environment also affects the integrity of the bonded complex. In the oral cavity, water can promote degradation of resin-based materials by weakening the filler–matrix interface, facilitating filler loss and plasticizing the resin matrix. These changes may increase stress concentration within the material and accelerate crack development. Thermal stress may further compromise the interfaces between root dentin and the core material, as well as between the core and the pretreated post. Under the combined effects of moisture and thermal cycling, interfacial deterioration and microleakage may occur, which can ultimately reduce frictional retention and impair the performance of the resin core build-up material [4].

### 4.5. Versatility of Universal Adhesives

Unlike the root dentin substrate, which had a uniform composition (taking into consideration the differences that exist between individuals), each post presented a different composition, as seen in Table 3. There were differences in terms of post material (quartz fiber vs. glass fiber), matrix composition, and, hence, polymerization contraction stress—which is dependent on the chemical composition of the resin matrix and which affects interfacial bonding. Apart from the fiber post, bonding performance is also affected by the universal adhesive and filler content of the core build-up material [18].

In this study, the bonding performance results in Table 4 and Table 5 were not only clinically acceptable, but remained stable and consistent from the Base time period to the TC 20k time period. Therefore, the second hypothesis was fully accepted because the universal adhesives could provide durable bonding between root dentin, fiber post and core build-up material, and this bonding was not influenced by the time period.

It is noteworthy that the results on the universal adhesives in this study highlighted their contribution to the treatment of traumatized teeth. Not only are they easy to use and convenient, but universal adhesives could also provide good bonding results across different substrates and interfaces, which augur well for the success and longevity of dental restorations.

### 4.6. Applicability of Tokuyama Universal Bond II as General-Purpose Universal Adhesive

When Tokuyama Universal Bond II was used as a pretreatment agent for post–core systems from other manufacturers, the resulting bond strength was generally comparable to, and in many cases higher than, that obtained with the manufacturer-recommended pretreatment agents (Table 5 and Table 6). These findings suggest that this two-bottle adhesive system can provide stable and durable bonding between fiber posts and core build-up materials.

One possible explanation for this performance is the formulation of two-bottle pretreatment systems. In such systems, premature hydrolysis of the silane coupling component before use is less likely to occur. Instead, hydrolysis proceeds after the two components are mixed, leading to the generation of silanol groups at the time of application. This mechanism may increase the availability of reactive silanol groups for bonding to the fiber post surface, thereby contributing to stronger chemical interaction and improved retention during durability testing [18,19]. This interpretation is supported by the SEM findings, which showed firm adhesion of the core build-up material to the fiber post surface, with penetration into the fiber structure (Figure 2).

Among the post–core systems tested, ESTECEM II Plus/Tokuyama FR Post showed the highest pull-out force values at both evaluation periods, and these values were significantly higher than those of the other systems. This superior performance may be explained by two factors. First, the core material exhibited higher flexural strength and flexural modulus of elasticity than the other materials tested. Second, pretreatment of the post surface with Tokuyama Universal Bond II appears to have enhanced post retention, as discussed above [18,19].

The adhesive performance of Tokuyama Universal Bond II may also be related to its functional monomer, the New 3D-SR monomer, which has been associated with storage stability at room temperature and resistance to hydrolysis or degradation of γ-MPTES [3-(triethoxysilyl)propyl methacrylate]. Within the limits of the present study, these results suggest that the application of Tokuyama Universal Bond II may extend beyond the manufacturer-recommended indications and that it may serve as a general-purpose pretreatment agent for bonding between fiber posts and core build-up materials. On this basis, the third hypothesis was accepted.

### 4.7. Limitations

This study presents results obtained under limited in vitro conditions. In fact, the materials used in this study are actually employed in the oral cavity. Further measurements simulating thermal changes and occlusal forces within the oral environment are necessary.

## 5. Conclusions

Universal adhesives were not only practical for use with a variety of substrates but also effective in establishing strong bonding among root canal dentin, resin core build-up materials, and fiber posts. In addition, this bonding performance remained stable after thermocycling, as no significant differences were detected between the Base and TC 20k conditions.

## Figures and Tables

**Table 1:** Core build-up materials used in this study.

Product	Composition	Manufacturer	Batch No.
RelyX Universal Resin Cement	Surface-treated glass powder filler, Phosphate ester monomer, TEGDMA, DiurethaneDimethacrylate, Silica filler, initiator, Titanium Dioxide.	Solventum, Seefeld, Germany	11531204
Clearfil DC Core Automix ONE	Bis-GMA, TEGDMA, Hydrophilic aliphatic dimethacrylate, Hydrophobic aromatic dimethacrylate, Silanated barium glass filler, Silanated colloidal silica, Colloidal silica, dl-Camphor Quinone, Aluminum oxide filler, initiators, Accelerators, Pigments. Filler content: 74 wt.%, 52 vol.%.	Kuraray Noritake Dental, Tainai, Japan	8L06384
ESTECORE	Bis-GMA, TEGDMA, Bis-MPEPP, Silica–Zirconia Filler, Camphorquinone, Peroxide, Radial amplifier, others. Filler content: 75 wt.%.	Tokuyama Dental, Tokyo, Japan	U295
ESTECEM II Plus	Paste A: Bis-GMA, TEGDMA, Bis-MPEPP, Silica–Zirconia Filler. Paste B: Bis-GMA, TEGDMA, Bis-MPEPP, Silica–Zirconia Filler, Camphorquinone, Peroxide. Filler content: 74 wt.%.	Tokuyama Dental, Tokyo, Japan	A09719
UniFil Core EM	UDMA, dimethacrylate, Fluoroaluminosilicate glass, Iron oxide, Dibenzoyl peroxide, Butylated hydroxytoluene. Filler content: 75 wt.%.	GC, Hasunuma, Itabashi, Japan	2302201
MultiCore Flow	Ytterbium trifluoride, Bis-GMA, UDMA, TEGDMA, Dibenzoyl peroxide. Filler content: 70 wt.%, 46 vol.%. The particle size ranges from 0.04 to 25 µm.	Ivoclar Vivadent AG, Schaan, Liechtenstein	ZO7ZCY
BeaitiLink SA	Paste A: Zirconium silicate filler, Bis-GMA, Phosphonic acid monomer, Carboxylic acid monomer, Polymerization initiator, others. Paste B: Glass powder filler (S-PRG filler), UDMA, Polymerization initiator, Pigments, others. Filler content: approximately 60 wt.%.	Shofu, Kyoto, Japan	122303
NX3	Barium Aluminoborosilicate glass, Ytterbium trifluoride, Fumed Silica, TEGDMA, UDMA, EBPADMA, initiator, Stabilizer. Filler content: 67.5 wt.%, 43.3 vol.%.	Kerr, Orange, CA, USA	A172592
Core-X flow	Urethane dimethacrylate, Di- & Tri-functional Methacrylates, Barium Boron, Fluoroaluminosilicate glass, Camphorquinone (CQ) Photoinitiator, Photoaccelerators, Silicon Dioxide, Benzoyl Peroxid.	DENTSPLY Caulk, Milford, DE, USA	DE 19963
SI-300381	Paste A: Fluoroboroaluminosilicate glass, Bis-GMA, TEGDMA, Polymerization initiator, Pigments, others. Paste B: Fluoroboroaluminosilicate glass, Bis-GMA, TEGDMA, Polymerization initiator, others.	Shofu, Kyoto, Japan	250517D
i-TFC system Post Resin	Dimethacrylates, Silica, Barium glass filler, Photoinitiators, Stabilizer, others. Filler content: 67 wt.%.	SUN MEDICAL, Moriyama, Japan	MX13

TEGDMA: triethyleneglycol dimethacrylate, Bis-GMA: bisphenol A diglycidylmethacrylate.

**Table 2:** Universal adhesives used in this study.

Adhesive	Batch No.	Composition	Manufacturer	Surface Treatment
Scotchbond Universal Plus Adhesive	8846013	Brominated dimethacrylate, HEMA, Silane Treated Silica, Vitrabond Copolymer, MDP, initiators, MPTES, Ethanol, water	3M, Seefeld, Germany	Scotchbond Universal Plus Adhesive (20 s)–air (5 s)
CLEAFIL Universal Bond *Quick 2*	AH0023	3-Methacryloxypropyl trimethoxysilane, MDP, Ethanol	Kuraray Noritake Dental, Tainai, Japan	CLEAFIL CERAMIC PRIMER PLUS (1–2 s)–air (5 s)
G-Cem One Multi Primer	2104221	Vinyl silane, Phosphate ester monomer, Thiophosphate ester monomer, Methacrylic ester, Ethanol	GC, Hasunuma, Itabashi, Japan	G-Cem One Multi Primer (10 s)–air (5 s)
Tokuyama Universal Bond II (A + B)	Bond A: 0011 Bond B: 0510	**Liquid A**: Phosphoric acid monomer (New 3D-SR monomer), MTU-6, HEMA, Bis-GMA, TEGDMA, Acetone, others. **Liquid B**: γ-MPTES, Borate, Peroxide, Acetone, Ethanol, water, others	Tokuyama Dental, Tokyo, Japan	Tokuyama Universal Bond II Mix (Liquid A + Liquid B, 1–2 s)–air (5 s)
Monobond Plus	ZO1LG8	Phosphoric acid monomer, Silane methacylate, Ethanol	Ivoclar Vivadent AG, Schaan, Liechtenstein	Monobond Plus (60 s)–air
BeautiBond Xtreme	042347	Acetone, water, Bis-GMA, TEGDMA, Phosphoric ester monomer, Silane coupling agent, initiator, others	Shofu, Kyoto, Japan	BeautiBond Xtreme (20 s)–air
OptiBond eXTRa Universal	Primer: 8199022 Adhesive: 8181793	HEMA, dimethacrylate monomers, tri-functional methacrylate monomer, Ethanol, Photoinitiator, Bariumaluminosilicate filler, Silica, Sodium hexafluorosilicate	Kerr, Orange, CA, USA	OptiBond eXTRa Adhesive (15 s)–air (5 s)–LED light (5 s)
Prime & Bond universal	DE 19963	Phosphoric acid modified acrylate resin, Multifunctional acrylate, Bifunctional acrylate, Acidic acrylate, Isopropanol, water, initiator, Stabilizer	DENTSPLY Caulk, Milford, DE, USA	Endodontic post cementation Apply mixture of Prime & Bond universal & Dentsply Self-cure Activator post surface. Leave it undisturbed for 10 s and dry naturally. Post Cementation Put Core-X flow into the post space and immediately insert the post into the post space. Light cure from every direction using a light-curing unit to fix the post: Halogen for 20 s.
SI-303062	Primer A: 250317 Primer B: 250314	Primer A: Acetone, distilled water, Bis-GMA, Carboxylic acid monomer, TEGDMA, Phosphonic acid monomer, others Primer B: Distilled water, Acetone, initiator	Shofu, Kyoto, Japan	Preparation of the post Apply SHOFU Porcelain Primer to the post in one layer with a disposable brush. Leave it undisturbed for 10 s and dry naturally. Pretreatment of post space Use Primer A and Primer B in equal amounts and apply the mixture onto the entire adhesive surface of the post space. Leave undisturbed for 10 s to air dry with gentle air for 3 s and then dry with stronger air to dry the surface sufficiently. Light cure with a light-curing unit: Halogen for 10 s. Post Cementation Fill SI-300381 paste into the post space and immediately insert the post into the post space. Light cure from every direction using a light-curing unit to fix the post: Halogen for 20 s.
i-TFC Luminous Bond II	Bond: FW1, Catalyst blush: ES1	Bond: Methacrylic acid esters (4-META, others), Acetone, water, others Catalyst blush: Aromatic amines, aromatic sulfinates	SUN MEDICAL, Moriyama, Japan	Mix (Bond + Catalyst brush, 5 s)–air (10 s)–LED light (20 s)

MDP: 10-methacryloyloxydecyl dihydrogen phosphate, Bis-GMA: bisphenol A diglycidylmethacrylate, 4-MET: 4-methacryloxyethyl trimellitic acid, MTU-6: 6-methacryloxyhexyl 2-thiouracil-5-carboxylate, γ-MPTES: 3-(triethoxysilyl) propyl methacrylate.

**Table 3:** Fiber posts used in this study.

Product (Diameter)	Composition	Manufacturer	Batch No.
RelyX FiberPost (1.6 mm)	Glass fibers, composite resin matrix	Solventum, Seefeld, Germany	306831603
Tokuyama FR Post (1.6 mm)	Glass fibers, Colpolymer Bis-GMA resin	Tokuyama Dental, Tokyo, Japan	1604251
GC Fiber Post (1.6 mm)	Glass fibers, Metacrylate	GC, Tokyo, Japan	1609021
Clearfil AD Fiber Post (1.6 mm)	Glass fibers, Colpolymer Bis-GMA and Methacrylic acid monomer	Kuraray Noritake Dental, Tainai, Japan	7U0001
BeautiCore FiberPost (1.6 mm)	Glass fiber, Copolymer of Bis-GMA and Methacrylic ester monomer	Shofu, Kyoto, Japan	41601
i-TFC Luminous Fiber II (1.6 mm)	Glass components: Borosilicate glass, barium oxide, and others Resin component: Dimethacrylate and diacrylate copolymer, others	SUN MEDICAL, Moriyama, Japan	EL1S

**Table 4:** Push-out force data of core build-up systems (N, mean (S.D.), Adh.).

Core Build-Up Systems: Resin Cement/Pretreating Agent/Fiber Post	Time		*t*-Test *
	Base (1 Day)	TC 20k	
RelyX Universal Resin Cement/ Scotchbond Universal Plus Adhesive/RelyX Fiber Post	19.8 (4.2, 0) ab ^#^	19.6 (3.1, 0) e	NS
Clearfil DC Core Automix ONE/Clearfil Universalbond Quick 2 + Clearfil Porcelain Bond Activator/Cleafil AD Fiber Post	24.5 (5.3, 0) abc	23.0 (4.3, 0) efgh	NS
UniFil Core EM/G-Premio BOND + G-Premio BOND DCA/GC Fiber Post	23.4 (4.6, 1) abc	20.4 (2.4, 0) ef	NS
ESTECEM II/ Tokuyama Universal Bond II (A + B)/Tokuyama FR Post	26.7 (3.6, 0) c	23.1 (3.9, 0) efgh	NS
ESTECORE Hand Type/ Tokuyama Universal Bond II (A + B)/Tokuyama FR Post	26.4 (2.9, 0) c	25.6 (3.2, 0) efg	NS
MultiCore Flow/Monobond Plus/BeautiCore Fiber	23.7 (5.0, 0) abc	18.9 (2.7, 0) e	S
NX3/OptiBond XTR + Porcelain Primer (Shofu)/BeautiCore Fiber	26.6 (4.9, 0) c	18.9 (3.2, 0) e	S
BeautiLink SA Automix + BeautiBond Xtreme, BeautiCore FiberPost	25.2 (3.6, 0) bc	24.1 (3.1, 0) efgh	NS
SI-303062 (A + B)/SI-300381/Post pretreated by BeautiBond Xtreme/BeautiCore FiberPost	28.7 (2.7, 0) d	28.0 (2.6, 0) h	NS
core-X flow/Prime & Bond universal + Self cure Activator/FluoroPost	25.5 (4.9,0) bc	22.6 (4.7, 0) efgh	NS
i-TFC system Post Resin/i-TFC Luminous Bond II/i-TFC Luminous Fiber II	21.0 (4.2, 0) abc	22.7 (4.2, 0) efgh	NS

*: S: significant difference (*p* < 0.05), NS: not significant difference (*p* > 0.05), ^#^: letters represent groups with no significant difference (a–h, Tukey HSD procedure), *p* > 0.05, TC 20k: after 20,000 thermocycles, *n* = 10, Adh: number of adhesive failure modes after failure.

**Table 5:** Pull-out force data between core build-up materials and fiber posts (N, mean (S.D.), Adh.).

Materials/Fiber Post (Each Manufacturer’s Recommended Pretreatment Agent)	Time		*t*-Test *
	Base (1 Day)	TC 20k	
RelyX Universal Resin Cement/RelyX Fiber Post (Scotchbond Universal Plus Adhesive)	30.5 (2.7, 0) abcde ^#^	25.6 (3.1, 0) jk	S
Clearfil DC Core Automix ONE/Clearfil AD Fiber Post (Clearfil Universalbond Quick 2)	30.5 (3.5, 0) abcde	30.6 (4.7, 0) jklno	NS
UniFil Core EM/GC Fiber Post (G-Premio BOND + G-Premio BOND DCA)	33.1 (5.0, 0) cdef	34.1 (5.0, 0) mnopq	NS
ESTECEM II/Tokuyama FR Post (Tokuyama Universal Bond II)	41.2 (3.4, 0) hi	41.8 (2.5, 0) s	NS
ESTECORE Hand Type/Tokuyama FR Post (Tokuyama Universal Bond II)	40.5 (3.7, 0) hi	44.3 (3.3, 0) s	NS
MultiCore Flow/BeautiCore Fiber (Monobond Plus)	36.5 (1.5, 0) ghi	23.6 (2.7, 0) j	S
NX3/BeautiCore Fiber (OptiBond XTR + Porcelain Primer)	40.2 (4.5, 0) ghi	27.9 (4.6, 0) jklmn	S
BeautiLink SA Automix/BeautiCore FiberPost (BeautiBond Xtreme)	32.9 (2.3, 0) bcdef	29.4 (3.9, 0) jklm	NS
SI-303062/BeautiCore FiberPost (BeautiBond Xtreme)	37.2 (3.5, 0) fghi	35.3 (3.3, 0) nopqr	NS
core-X flow/FluoroPost (Prime & Bond universal + Self cure Activator)	32.1 (4.1, 0) bcdef	26.9 (3.4, 0) jkl	NS
i-TFC system Post Resin/i-TFC Luminous Fiber II (i-TFC Luminous Bond II)	33.9 (4.0, 0) cdef	32.6 (5.0, 0) lmnop	NS

*: S: significant difference (*p* < 0.05), NS: not significant difference (*p* > 0.05), ^#^: letters represent groups with no significant difference (a–s, Tukey HSD procedure), *p* > 0.05, TC 20k: after 20,000 thermocycles, *n* = 10, Adh: number of adhesive failure modes after failure.

**Table 6:** Pull-out force data for pretreatment with Tokuyama Universal Bond II (N, mean (S.D.), Adh.).

Materials/Fiber Post (Pretreated by Tokuyama Universal Bond II)	Time		*t*-Test *
	Base (1 Day)	TC 20k	
RelyX Universal Resin Cement/RelyX Fiber Post	34.2 (4.5, 0) abcd ^#^	38.2 (4.7, 0) lmnopq	NS
Clearfil DC Core Automix ONE/Clearfil AD Fiber Post	34.7 (4.5, 0) abcde	35.5 (3.0, 0) jklmno	NS
UniFil Core EM/GC Fiber Post	38.0 (4.0, 0) bcdefgh	32.7 (2.8, 0) jkl	S
ESTECEM II/Tokuyama FR Post	41.2 (3.4, 0) fghi	41.8 (2.5, 0) pqr	NS
ESTECORE Hand Type/Tokuyama FR Post	40.5 (3.7, 0) efghi	44.3 (3.3, 0) r	NS
MultiCore Flow/BeautiCore FiberPost	42.5 (1.2, 0) hi	36.9 (5.5, 0) klmnopq	S
NX3/BeautiCore FiberPost	39.0 (2.6, 0) defghi	32.3 (1.7, 0) jkl	S
BeautiLink SA Automix/BeautiCore FiberPost	35.8 (4.1, 0) abcdefg	32.0 (3.1, 0) jk	NS
SI-303062/BeautiCore FiberPost	38.0 (4.0, 1) bcdefgh	35.0 (3.7, 0) jklmno	NS
core-X flow/FluoroPost	35.7 (2.9, 0) abcdef	32.8 (4.4, 0) jkl	NS
i-TFC system Post Resin/i-TFC Luminous Fiber II	33.2 (3.8, 0) abcd	30.1 (5.8, 0) j	NS

*: S: significant difference (*p* < 0.05), NS: not significant difference (*p* > 0.05), ^#^: letters represent groups with no significant difference (a–r, Tukey HSD procedure), *p* > 0.05, TC 20k: after 20,000 thermocycles, *n* = 10, Adh: number of adhesive failure modes after failure.

**Table 7:** Comparison of the means (*t*-Test) of pull-out force data between two pretreatment agents (manufacturer-recommended adhesive vs. Tokuyama Universal Bond II).

**RelyX Universal Resin Cement**		**Clearfil DC Core Automix ONE**		**UniFil Core EM**		**ESTECEM II**	
**Base**	**TC 20k**	**Base**	**TC 20k**	**Base**	**TC 20k**	**Base**	**TC 20k**
NS	S	NS	NS	NS	NS	NS	NS
**ESTECORE Hand Type**		**MultiCore Flow**		**NX3**		**BeautiLink SA Automix**	
**Base**	**TC 20k**	**Base**	**TC 20k**	**Base**	**TC 20k**	**Base**	**TC 20k**
NS	NS	NS	S	NS	S	NS	NS
**SI-303062**		**core-X flow**		**i-TFC system Post Resin**			
**Base**	**TC 20k**	**Base**	**TC 20k**	**Base**		**TC 20k**	
NS	NS	NS	S	NS		NS	

TC 20k: after 20,000 thermocycles, S: significant difference (*p* < 0.05), NS: not significant difference (*p* > 0.05).

**Table 8:** Shear bond strength data between root dentin and core build-up materials (MPa, mean (S.D.), Adh.).

Materials/Pretreating Agent	Time		*t*-Test *
	Base (1 Day)	TC 20k	
RelyX Universal Resin Cement/Scotchbond Universal Plus Adhesive	22.3 (3.9, 0) cd ^#^	19.2 (3.4, 0) fghi	NS
Clearfil DC Core Automix ONE/Clearfil Universalbond Quick 2	23.7 (3.7, 0) de	22.8 (2.9, 0) hij	NS
UniFil Core EM/G-Premio BOND + G-Premio BOND DCA	18.0 (4.1, 0) abc	19.0 (3.3, 0) fghi	NS
ESTECEM II/Tokuyama Universal Bond II	22.0 (2.4, 0) bcd	20.3 (2.0, 0) fghi	NS
ESTECORE Hand Type/Tokuyama Universal Bond II	22.5 (3.5, 0) cd	21.6 (2.2, 0) fghi	NS
MultiCore Flow/Monobond Plus	21.2 (2.8, 0) abcd	21.5 (3.3, 0) fghi	NS
NX3/OptiBond XTR	22.5 (3.0, 0) cd	16.8 (2.3, 0) fg	S
BeautiLink SA Automix/BeautiBond Xtreme	28.0 (4.3, 0) e	19.9 (2.4, 0) fghi	S
SI-303062/BeautiBond Xtreme	21.0 (2.6, 0) abcd	18.6 (1.9, 0) fghi	NS
core-X flow/Prime & Bond universal + Self cure Activator	20.7 (4.2, 0) abcd	18.1 (2.4, 0) fgh	NS
i-TFC system Post Resin/i-TFC Luminous Bond II	17.8 (2.9, 0) abc	16.4 (3.3, 0) f	NS

*: S: significant difference (*p* < 0.05), NS: not significant difference (*p* > 0.05), ^#^: letters represent groups with no significant difference (a–i, Tukey HSD procedure), *p* > 0.05, TC 20k: after 20,000 thermocycles, *n* = 10, Adh: number of adhesive failure modes after failure.

**Table 9:** Flexural strength data of core build-up materials (MPa, mean (S.D.)).

Materials	Time		*t*-Test *
	Base (1 Day)	TC 20k	
RelyX Universal Resin Cement	117.5 (7.0) c ^#^	108.8 (8.8) kl	NS
Clearfil DC Core Automix ONE	140.6 (9.6) efgh	140.9 (8.6) pqr	NS
UniFil Core EM	153.6 (11.4) i	151.2 (12.1) rs	NS
ESTECEM II	162.1 (11.7) i	146.4 (10.1) qr	S
ESTECORE Hand Type	172.2 (10.2) i	153.9 (13.3) rs	S
MultiCore Flow	142.1 (9.1) fgh	126.3 (8.2) mno	S
NX3	123.7 (9.8) cd	97.9 (8.7) k	S
BeautiLink SA Automix	128.9 (5.3) cdef	101.7 (9.3) k	S
SI-303062	133.3 (6.4) defg	132.2 (5.8) mnopq	NS
core-X flow	136.0 (9.7) defg	128.0 (10.6) mnop	NS
i-TFC system Post Resin	139.4 (6.4) efgh	143.6 (9.0) pqr	S

*: S: significant difference (*p* < 0.05), NS: not significant difference (*p* > 0.05), ^#^: letters represent groups with no significant difference (a–s, Tukey HSD procedure), *p* > 0.05, TC 20k: after 20,000 thermocycles, *n* = 10, Adh: number of adhesive failure modes after failure.

**Table 10:** Flexural modulus data of core build-up materials (GPa, mean (S.D.)).

Materials	Time		*t*-Test *
	Base (1 Day)	TC 20k	
RelyX Universal Resin Cement	4.27 (0.41) a ^#^	4.24 (0.28) i	NS
Clearfil DC Core Automix ONE	8.43 (0.55) cde	10.57 (1.10) nop	S
UniFil Core EM	11.12 (0.92) fg	11.26 (0.52) pq	NS
ESTECEM II	12.42 (1.79) g	10.72 (0.67) op	S
ESTECORE Hand Type	13.80 (1.35) h	12.52 (0.59) r	NS
MultiCore Flow	8.44 (0.47) cde	7.77 (0.81) kl	NS
NX3	5.97 (0.49) b	5.90 (0.46) j	NS
BeautiLink SA Automix	8.49 (0.95) cde	7.91 (0.86) kl	NS
SI-303062	8.97 (0.77) de	9.27 (0.96) mn	NS
core-X flow	8.86 (0.82) de	9.75 (0.77) mno	NS
i-TFC system Post Resin	7.35 (0.52) c	7.92 (0.35) kl	NS

*: S: significant difference (*p* < 0.05), NS: not significant difference (*p* > 0.05), ^#^: letters represent groups with no significant difference (a–r, Tukey HSD procedure), *p* > 0.05, TC 20k: after 20,000 thermocycles, *n* = 10, Adh: number of adhesive failure modes after failure.

**Table 11:** Correlations with push-out force in Table 4 (*n* = 22).

vs. Table 4	*r*	*p*
Table 5	0.656	0.0009
Table 8	0.343	0.112
Table 9	0.409	0.058
Table 10	0.475	0.025

## Data Availability

The data presented in this study are available from the corresponding author, M.I., upon reasonable request.
